# Multimodal assessment of fetal head circumference and biparietal diameter: systematic differences between MRI and US measurements and development of an US-equivalent prediction regression model

**DOI:** 10.3389/fradi.2026.1828351

**Published:** 2026-07-14

**Authors:** Kai Ye, Kexin Wang, Pengsheng Wu, Xiaoying Wang

**Affiliations:** 1 Department of Radiology, Peking University First Hospital, Beijing, China; 2 Beijing Smart Tree Medical Technology Co., Ltd., Beijing, China

**Keywords:** biparietal diameter, fetal magnetic resonance imaging, gestational age, head circumference, linear regression, ultrasound

## Abstract

**Objective:**

To investigate the differences between fetal magnetic resonance imaging (MRI) and ultrasonography (US) in measuring head circumference (HC) and biparietal diameter (BPD), and to develop and validate a regression model for predicting US-equivalent values based on MRI measurements.

**Methods:**

Imaging data of 124 mid-to-late trimester fetuses were retrospectively included. The differences in HC and BPD measurements between the two modalities were quantitatively analyzed. A regression model was constructed using MRI measurements, gestational age (GA), and the time interval between examinations as independent variables to predict the US-equivalent values (USpred).

**Results:**

Compared with US, MRI demonstrated significant systematic overestimation for both HC (mean difference: 14.0 mm, *p* < 0.001) and BPD (median difference: 3.18 mm, *p* < 0.001). The established USpred model showed high goodness-of-fit (adjusted *R*^2^ > 0.95). When classifying based on US standards, the USpred values exhibited significantly improved classification efficacy compared to direct MRI measurements: overall accuracy (HC: 0.782 vs. 0.629; BPD: 0.863 vs. 0.782) and Matthews correlation coefficient (HC: 0.512 vs. 0.322; BPD: 0.639 vs. 0.528).

**Conclusion:**

Fetal MRI measurements of HC and BPD show a systematic overestimation compared to US. The USpred model can effectively correct for this systematic bias in MRI measurements and the influence of examination time intervals, significantly improving agreement with commonly used clinical US standards. It provides a reliable tool for the precise assessment of prenatal MRI.

## 1 Background

Fetal central nervous system abnormalities are among the most common types of congenital anomalies (1) which include abnormalities in fetal head development. Macrencephaly or fetal macrosomia can adversely affect both maternal and neonatal outcomes, increasing the risk of cesarean delivery, postpartum hemorrhage, severe neurological dysfunction, and even neonatal death (2); while microcephaly is often associated with restricted fetal growth and development (3, 4). Therefore, prenatal imaging plays a critical role in monitoring fetal development (5, 6).

Owing to its non-invasive nature, absence of radiation, and real-time imaging capabilities, ultrasonography (US) has become the primary imaging modality for prenatal screening (7–9). However, its diagnostic efficacy is somewhat operator-dependent and can be affected by factors such as maternal obesity, polyhydramnios, and increased fetal skull calcification in the third trimester (10–12). Fetal magnetic resonance imaging (MRI), recognized for its excellent tissue contrast, multiplanar imaging capability, high spatial resolution, and lower operator dependence, is being increasingly utilized in clinical practice (13–15). It is particularly valuable for providing additional diagnostic information when anomalies are suspected on US (16). Numerous studies have affirmed the significant value of MRI in assessing the growth and development of various fetal organ systems (14, 17).

Head circumference (HC) and biparietal diameter (BPD) are crucial biometric parameters for evaluating fetal growth. Current clinical practice primarily relies on US-derived reference ranges for HC and BPD across different gestational weeks, which serve as the standard for assessing fetal development. Nevertheless, skull ossification in the late stages of pregnancy can compromise the accuracy of US measurements. In this context, MRI emerges as a valuable complementary tool, demonstrating higher reliability for quantitative assessment (17, 18). Although several studies have endeavored to establish MRI-based reference values for fetal biometry (19–21), a unified standard for gestational age-specific HC and BPD measurements using MRI has not yet been established. There is a particular lack of systematic research correlating MRI measurements with US values. While the study by E. Katorza et al. explored the correlation of fetal vermis parameters between MRI and US, but their analysis did not perform intra-individual level comparisons within the same subjects over a short time interval. Furthermore, comprehensive multimodal comparative studies focusing specifically on HC and BPD remain scarce (22, 23).

This study aims to systematically analyze the agreement, differences, and correlation between US and MRI measurements of fetal HC and BPD at comparable gestational ages, and to explore their clinical implications. Addressing the practical reality of a time interval between these two examinations in clinical settings, we intend to construct regression models to adjust for the effects of this time difference and to evaluate the classification performance of both this model and MRI in determining abnormal fetal head development. This will enable an assessment of the consistency between their classification results and US outcomes, as well as their clinical applicability.

## 2 Materials and methods

### 2.1 Study design and ethical approval

This retrospective study was approved by the Hospital Ethics Committee of Peking University First Hospital, Approval No. [2023–858]. The requirement for written informed consent was waived because of the retrospective nature of the study.

### 2.2 Data inclusion

Fetal MR images and related clinical and US data from the year 2020 were retrospectively retrieved from the PACS system of Peking University First Hospital.

The inclusion criterion was as follows: patients who underwent fetal nervous system MRI at Peking University First Hospital.

The exclusion criteria were as follows:

1. Lack of a definitive imaging diagnosis or missing imaging/US reports;
2. Substandard MR image quality;
3. Non-singleton pregnancies;
4. An interval exceeding 7 days between the US and MR examinations.

After applying the above criteria for image selection, the final model training cohort comprised 124 examinations from 124 patients. Among these, 96 patients underwent US first, while 28 underwent MR first.

### 2.3 Overall workflow and system pipeline

The overall workflow of this study consisted of five major stages: data acquisition, image processing, biometric measurement, regression modeling, and classification evaluation.

First, eligible fetal MRI and corresponding US records were retrospectively retrieved from the institutional PACS and clinical reporting system. US-derived HC and BPD values, gestational age at US examination, and examination dates were extracted from clinical US reports. MRI examination date and MRI-based gestational age were recorded from radiological records.

Second, all fetal MRI datasets were uploaded into the ITK-based image-processing platform. A cranial cavity segmentation model was used to automatically identify the fetal cranial cavity on axial T2-weighted images. The slice with the largest cranial cross-sectional area was automatically selected as the standard measurement plane.

Third, on the selected MRI slice, a junior radiologist manually delineated the cranial bone contour, assisted by the segmentation output. The delineation was then reviewed and confirmed by a senior radiologist to ensure measurement reliability. After contour confirmation, preset algorithms automatically calculated HC by tracing the outer cranial bone contour and measured BPD as the distance from the outer table of one parietal bone to the inner table of the opposite parietal bone.

Fourth, paired MRI and US measurements were compared at the individual level. Measurement differences were calculated as MRI—US. The influence of examination sequence and gestational-age interval was also assessed.

Fifth, linear regression models were developed to predict US-equivalent HC and BPD values from MRI-derived measurements. The predicted values, referred to as USpred, were then classified according to US-based reference standards and compared with the actual US classifications. Direct MRI-based classifications were also compared with US classifications to evaluate whether USpred improved clinical agreement.

**Figure 1:**
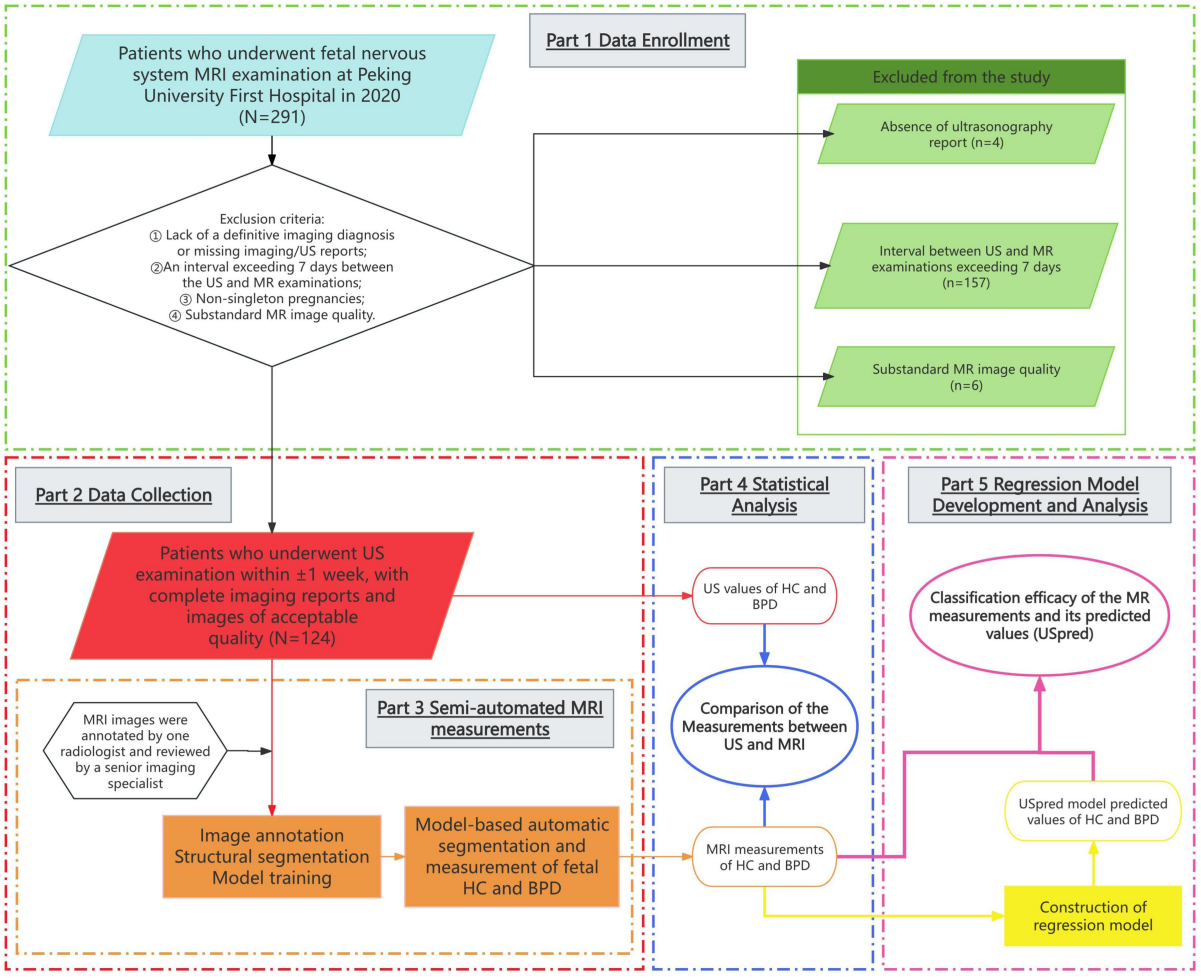
Study workflow and system pipeline. The workflow consisted of data retrieval, eligibility screening, US report extraction, MRI image upload, cranial cavity segmentation, maximal cross-sectional slice selection, cranial bone contour delineation, automatic HC/BPD measurement, paired MRI–US comparison, US-equivalent regression modeling, model diagnostics, and classification performance evaluation.

This workflow is illustrated in Figure 1.

### 2.4 MR scanning protocol

The MRI image data in this study were acquired using scanners from two manufacturers: Philips and Siemens, employing magnetic field strengths of both 1.5 T and 3.0 T across three scanner models: Achieva, Aera, and Magnetom Vida. The images were obtained using a slice thickness of 5.00 [4.00, 5.00] mm [median (interquartile range)] and a slice gap of 5.00 [4.80, 5.00] mm.

### 2.5 US and MRI measurement of HC and BPD

US measurements were retrospectively extracted from routine clinical imaging reports. MRI measurements were performed using a semi-automated method. After all cases were collected, MRI images were uploaded into the ITK platform. The cranial cavity segmentation model automatically detected the fetal cranial region and selected the slice with the maximum cross-sectional area. A junior radiologist manually delineated the cranial bone contour on this selected slice. The final contour was reviewed by a senior radiologist.

**Figure 2:**
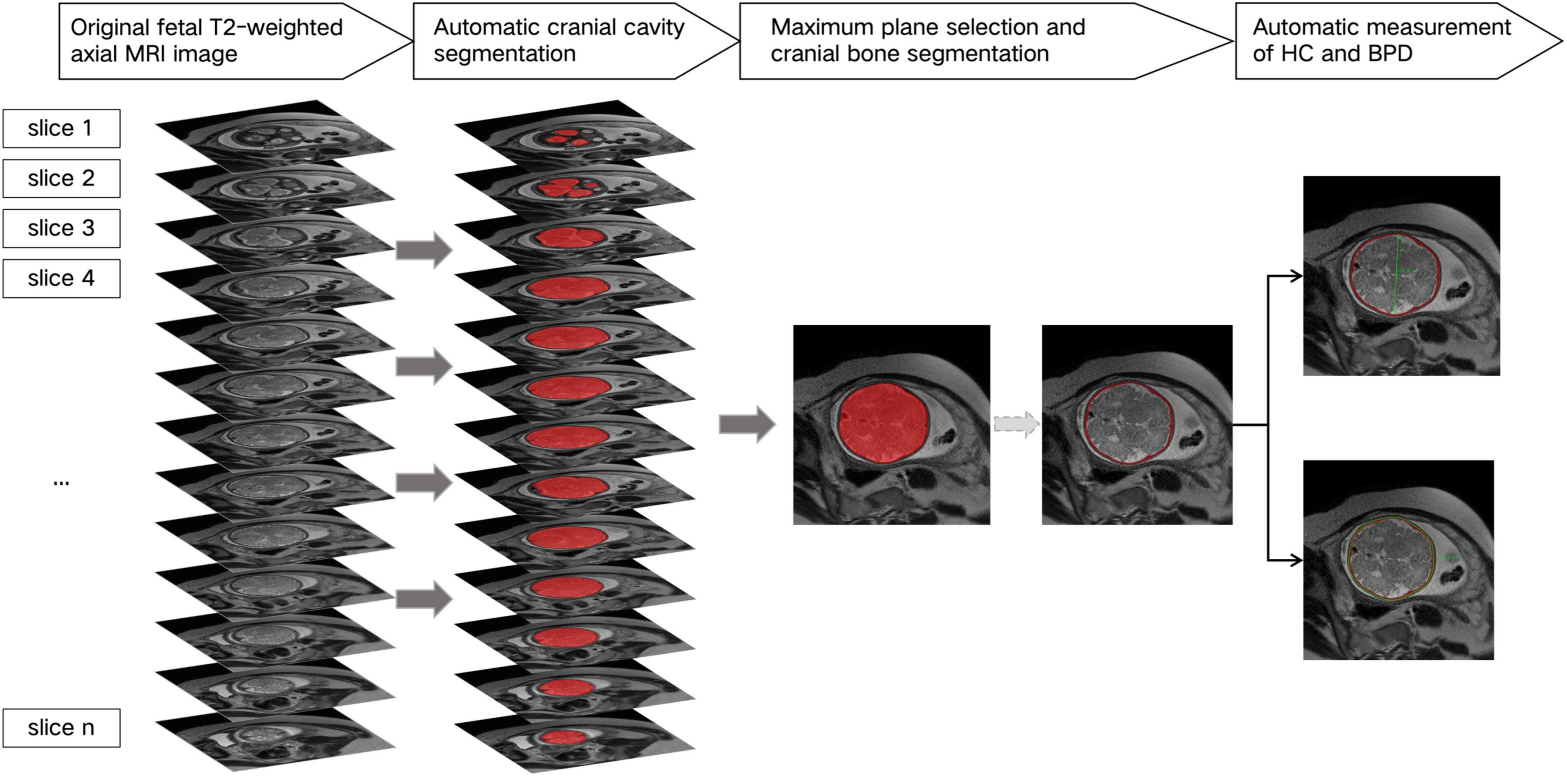
MRI segmentation and biometric measurement procedure. Step 1: input of fetal T2-weighted axial MRI image. Step 2: automatic cranial cavity segmentation. Step 3: automatic selection of the slice with the largest cranial cross-sectional area and cranial bone contour delineation. Step 4: automatic measurement of HC and BPD.

HC was calculated by tracing the outer contour of the cranial bones. BPD was defined as the distance from the outer table of one parietal bone to the inner table of the opposite parietal bone. The MRI segmentation and measurement procedure is shown in Figure 2.

### 2.6 Qualitative classification using US-based references

Because no universally accepted MRI-based reference standard for fetal HC and BPD is currently available, this study used the US reference values for fetal biological parameters published by the Chinese Medical Association (24) as the classification criterion for both US, direct MRI, and USpred measurements.

For fetuses between 25 and 40 weeks of gestation, HC and BPD were classified into three categories:

1. Large: measurement greater than the 97th percentile;
2. Small: measurement lower than the 3rd percentile;
3. Normal: measurement between the 3rd and 97th percentiles.

US classification was used as the reference standard for evaluating the classification performance of direct MRI measurements and USpred values.

### 2.7 Comparison of the measurements between US and MRI

HC and BPD measurements obtained from US and MRI were compared in the entire cohort and further stratified according to examination sequence: USFirst and MRFirst. Descriptive statistics were calculated for gestational age, HC, and BPD. Continuous variables were summarized as mean ± standard deviation or median with interquartile range, depending on data distribution. Measurement differences were calculated as MRI–US. Between-group comparisons were performed using independent-samples *t*-tests for normally distributed data or Mann–Whitney *U*-tests for non-normally distributed data.

### 2.8 Correlation analysis

Pearson correlation coefficients were calculated to assess the linear relationships between gestational age and biometric parameters, including US-HC, MRI-HC, US-BPD, and MRI-BPD. Correlation coefficients and corresponding *P* values were reported.

### 2.9 Development of US-equivalent prediction models

Two linear regression models were developed to predict US-equivalent HC and BPD values from MRI measurements. The independent variables included the corresponding MRI measurement, MRI gestational age, GA_MR, the gestational-age difference between MRI and US, GA_Diff, and the interaction term GA_MR × GA_Diff. GA_Diff was defined as follows:

GA_Diff=GA_MR−GA_US

The HC prediction model was defined as:

$$\begin{array}{rl} HC\_USpred & =\beta_{0}+\beta_{1}\cdot HC\_MR+\beta_{2}\cdot GA\_MR+\beta_{3}\cdot GA\_Diff\\ & \;+\beta_{4}\cdot (GA\_MR\times GA\_Diff)+\varepsilon \end{array}$$

The BPD prediction model was defined as:

$$\begin{array}{rl} BPD\_USpred & =\alpha_{0}+\alpha_{1}\cdot BPD\_MR+\alpha_{2}\cdot GA\_MR+\alpha_{3}\cdot GA\_Diff\\ & \;+\;\alpha_{4}\cdot (GA\_MR\times GA\_Diff)+\varepsilon \end{array}$$

Model goodness of fit was assessed using adjusted *R*^2^, *F*-statistics, and *P* values. Regression coefficients, standard errors, and *P* values were reported for each predictor.

### 2.10 Model diagnostics

Regression assumptions were evaluated using residual diagnostic tests. The Shapiro–Wilk test was used to assess residual normality. The Breusch–Pagan test was used to evaluate homoscedasticity. The Durbin–Watson test was used to detect first-order autocorrelation. The RESET test was applied to examine model specification. Variance inflation factors, VIFs, were calculated to evaluate multicollinearity. Diagnostic plots were generated for visual assessment of residual distribution, fitted values, and influential observations.

### 2.11 Classification performance evaluation

Direct MRI measurements and USpred values were classified into large, normal, or small categories using the same US-based reference standards. These classifications were compared with actual US-based classifications. Classification performance was evaluated using overall accuracy, sensitivity, specificity, positive predictive value, negative predictive value, F1 score, Matthews correlation coefficient, MCC, and Cohen's Kappa. Performance metrics were calculated for HC and BPD separately.

### 2.12 Statistical analysis

Statistical analyses were performed using R software, version 4.1. A two-sided *P* value less than 0.05 was considered statistically significant. Continuous variables were reported as mean ± standard deviation or median with interquartile range. Categorical variables were reported as counts and percentages. Group comparisons were conducted using *t*-tests, Mann–Whitney *U*-tests, chi-square tests, or Fisher's exact tests, as appropriate.

## 3 Results

### 3.1 Difference of the HC and BPD measured by US and MRI

Table 1 shows the differences in HC and BPD measured by US and MRI. A total of 124 fetuses were included, with 96 undergoing ultrasound first (USFirst) and 28 undergoing MRI first (MRFirst). GA of US and MR was highly consistent, with a median difference of 0.286 weeks and no significant effect of exam sequence. For BPD, MR measurements were slightly larger than US, with a median MR–US difference of 3.18 mm (*P* < 0.001). Fetuses in the MRFirst group were more frequently classified as having large BPD compared with the USFirst group (35.7% vs. 10.4%, *P* = 0.006). Similarly, HC was consistently larger on MR than on US, with a mean MR–US difference of 14.0 mm (*P* < 0.001). The proportion of fetuses classified as large HC was significantly higher in the USFirst group compared with the MRFirst group (12.5% vs. 39.3%, *P* = 0.005).

**Table 1:** Demographic and measurement differences between US and MRI according to examination sequence.

Measurements	Overall (*N* = 124)	USFirst (*N* = 96)	MRFirst (*N* = 28)	*p*
US measurement				
GA_US (week)				
Mean (SD)	31.4 (3.58)	31.2 (3.78)	31.8 (2.80)	0.804
BPD_US (mm)				
Median [Q1,Q3]	81.7 [73.5,86.1]	80.8 [71.2,86.3]	83.2 [77.2,85.9]	0.232
BPD_US_Percentage				
<3rd	10 (8.1%)	8 (8.3%)	2 (7.1%)	0.061
3rd–5th	4 (3.2%)	4 (4.2%)	0 (0%)	
5th–10th	4 (3.2%)	4 (4.2%)	0 (0%)	
10th–50th	28 (22.6%)	22 (22.9%)	6 (21.4%)	
50th–90th	42 (33.9%)	36 (37.5%)	6 (21.4%)	
90th–95th	9 (7.3%)	6 (6.3%)	3 (10.7%)	
95th–97th	7 (5.6%)	6 (6.3%)	1 (3.6%)	
≥97th	20 (16.1%)	10 (10.4%)	10 (35.7%)	
BPD_US_Class				
Large	20 (16.1%)	10 (10.4%)	10 (35.7%)	0.006
Normal	94 (75.8%)	78 (81.3%)	16 (57.1%)	
Small	10 (8.1%)	8 (8.3%)	2 (7.1%)	
HC_US (mm)				
Median [Q1,Q3]	295 [271,309]	292 [266,311]	298 [281,306]	0.398
HC_US_Percentage				
<3rd	16 (12.9%)	14 (14.6%)	2 (7.1%)	0.003
3rd–5th	3 (2.4%)	3 (3.1%)	0 (0%)	
5th–10th	6 (4.8%)	2 (2.1%)	4 (14.3%)	
10th–50th	27 (21.8%)	25 (26.0%)	2 (7.1%)	
50th–90th	39 (31.5%)	32 (33.3%)	7 (25.0%)	
90th–95th	7 (5.6%)	5 (5.2%)	2 (7.1%)	
95th–97th	3 (2.4%)	3 (3.1%)	0 (0%)	
≥97th	23 (18.5%)	12 (12.5%)	11 (39.3%)	
HC_US_Class				
Large	23 (18.5%)	12 (12.5%)	11 (39.3%)	0.005
Normal	85 (68.5%)	70 (72.9%)	15 (53.6%)	
Small	16 (12.9%)	14 (14.6%)	2 (7.1%)	
MR measurement				
GA_MR (week)				
Mean (SD)	31.6 (3.53)	31.7 (3.75)	31.3 (2.73)	0.327
BPD_MR (mm)				
Median [Q1,Q3]	85.3 [76.3,89.4]	85.6 [74.9,89.9]	85.0 [79.3,87.2]	0.851
HC_MR (mm)				
Median [Q1,Q3]	306 [280,325]	308 [275,327]	304 [288,319]	0.470
Difference between MR and US (MR - US)				
GA_Diff (week)				
Median [Q1,Q3]	0.286 [0,0.571]	0.286 [0.143,0.714]	−0.429 [−0.857,−0.286]	/
absGA_Diff (week)				
Median [Q1,Q3]	0.429 [0.143,0.714]	0.286 [0.143,0.714]	0.429 [0.250,0.714]	0.115
HC_Diff (mm)				
Mean (SD)	14.0 (8.49)	16.2 (7.44)	6.36 (7.40)	<0.001
BPD_Diff (mm)				
Median [Q1,Q3]	3.18 [1.84,4.94]	3.80 [2.28,5.59]	1.61 [0.730,2.39]	<0.001

When further stratified by US-based classifications of HC and BPD (Table 2), MRI continued to yield systematically larger values across all subgroups. The mean HC difference (MR–US) remained positive in fetuses classified as large, normal, or small by US (8.0, 15.0, and 17.4 mm, respectively; *P* = 0.259), and the median BPD difference (MR–US) was similarly positive across subgroups (large: 2.25 mm, normal: 3.30 mm, small: 3.40 mm; *P* = 0.044). These findings indicate that MRI systematically overestimates HC and BPD compared with US, regardless of fetal head size category.

**Table 2:** MRI–US measurement differences stratified by US-based fetal head-size classification.

Measurements	Classification according to HC				Classification according to BPD			
	Large	Normal	Small	*p*	Large	Normal	Small	*p*
	(*N* = 23)	(*N* = 85)	(*N* = 16)		(*N* = 20)	(*N* = 94)	(*N* = 10)	
US measurement								
GA_US (week)								
Mean (SD)	31.4 (2.64)	31.2 (3.81)	32.3 (3.57)	0.505	32.0 (2.72)	31.2 (3.81)	31.8 (2.93)	0.768
BPD_US (mm)								
Median [Q1,Q3]	85.8 [83.0,92.4]	80.8 [73.5,85.8]	76.2 [66.9,80.0]	<0.001	91.4 [83.8,93.9]	81.2 [73.5,85.0]	70.8 [64.6,77.6]	<0.001
BPD_US_Percentage								
<3rd	0 (0%)	1 (1.2%)	9 (56.3%)	<0.001	0 (0%)	0 (0%)	10 (100%)	<0.001
3rd–5th	0 (0%)	1 (1.2%)	3 (18.8%)		0 (0%)	4 (4.3%)	0 (0%)	
5th–10th	0 (0%)	2 (2.4%)	2 (12.5%)		0 (0%)	4 (4.3%)	0 (0%)	
10th–50th	0 (0%)	26 (30.6%)	2 (12.5%)		0 (0%)	28 (29.8%)	0 (0%)	
50th–90th	0 (0%)	42 (49.4%)	0 (0%)		0 (0%)	42 (44.7%)	0 (0%)	
90th–95th	6 (26.1%)	3 (3.5%)	0 (0%)		0 (0%)	9 (9.6%)	0 (0%)	
95th–97th	3 (13.0%)	4 (4.7%)	0 (0%)		0 (0%)	7 (7.4%)	0 (0%)	
≥97th	14 (60.9%)	6 (7.1%)	0 (0%)		20 (100%)	0 (0%)	0 (0%)	
BPD_US_Class								
Large	14 (60.9%)	6 (7.1%)	0 (0%)	<0.001	20 (100%)	0 (0%)	0 (0%)	<0.001
Normal	9 (39.1%)	78 (91.8%)	7 (43.8%)		0 (0%)	94 (100%)	0 (0%)	
Small	0 (0%)	1 (1.2%)	9 (56.3%)		0 (0%)	0 (0%)	10 (100%)	
HC_US (mm)								
Median [Q1,Q3]	307 [301,325]	292 [265,308]	279 [253,289]	<0.001	318 [299,328]	292 [268,306]	269 [245,285]	<0.001
HC_US_Percentage								
<3rd	0 (0%)	0 (0%)	16 (100%)	<0.001	0 (0%)	7 (7.4%)	9 (90.0%)	<0.001
3rd–5th	0 (0%)	3 (3.5%)	0 (0%)		0 (0%)	3 (3.2%)	0 (0%)	
5th–10th	0 (0%)	6 (7.1%)	0 (0%)		0 (0%)	6 (6.4%)	0 (0%)	
10th–50th	0 (0%)	27 (31.8%)	0 (0%)		0 (0%)	26 (27.7%)	1 (10.0%)	
50th–90th	0 (0%)	39 (45.9%)	0 (0%)		2 (10.0%)	37 (39.4%)	0 (0%)	
90th–95th	0 (0%)	7 (8.2%)	0 (0%)		3 (15.0%)	4 (4.3%)	0 (0%)	
95th–97th	0 (0%)	3 (3.5%)	0 (0%)		1 (5.0%)	2 (2.1%)	0 (0%)	
≥97th	23 (100%)	0 (0%)	0 (0%)		14 (70.0%)	9 (9.6%)	0 (0%)	
HC_US_Class								
Large	23 (100%)	0 (0%)	0 (0%)	<0.001	14 (70.0%)	9 (9.6%)	0 (0%)	<0.001
Normal	0 (0%)	85 (100%)	0 (0%)		6 (30.0%)	78 (83.0%)	1 (10.0%)	
Small	0 (0%)	0 (0%)	16 (100%)		0 (0%)	7 (7.4%)	9 (90.0%)	
MR measurement								
GA_MR (week)								
Mean (SD)	31.4 (2.67)	31.4 (3.71)	32.7 (3.62)	0.381	31.8 (2.75)	31.5 (3.75)	32.1 (3.02)	0.952
BPD_MR (mm)								
Median [Q1,Q3]	91.7 [85.3,95.2]	85.0 [75.9,89.0]	80.6 [71.1,84.3]	<0.001	93.1 [85.1,96.4]	85.4 [76.0,88.6]	75.5 [69.1,80.7]	<0.001
HC_MR (mm)								
Median [Q1,Q3]	324 [306,330]	305 [275,324]	300 [274,306]	0.003	327 [306,333]	305 [275,321]	285 [267,304]	0.001
Difference between MR and US (MR - US)								
GA_Diff (week)								
Median [Q1,Q3]	0 [−0.429,0.286]	0.286 [0,0.571]	0.500 [0.143,0.857]	0.007	-0.071 [−0.464,0.143]	0.286 [0,0.714]	0.286 [0.036,0.821]	0.001
absGA_Diff (week)								
Median [Q1,Q3]	0.429 [0.143,0.714]	0.286 [0.143,0.714]	0.714 [0.250,0.857]	0.259	0.286 [0.143,0.571]	0.429 [0.143,0.714]	0.571 [0.286,0.821]	0.449
HC_Diff (mm)								
Mean (SD)	8.04 (8.67)	15.0 (7.91)	17.4 (7.57)	<0.001	9.55 (6.84)	14.7 (8.63)	16.8 (7.63)	0.014
BPD_Diff (mm)								
Median [Q1,Q3]	2.26 [1.09,3.65]	3.28 [2.16,5.20]	3.39 [1.51,4.71]	0.096	2.25 [0.997,3.41]	3.30 [2.04,5.17]	3.40 [1.75,4.58]	0.044

### 3.2 HC and BPD across GA

**Figure 3:**
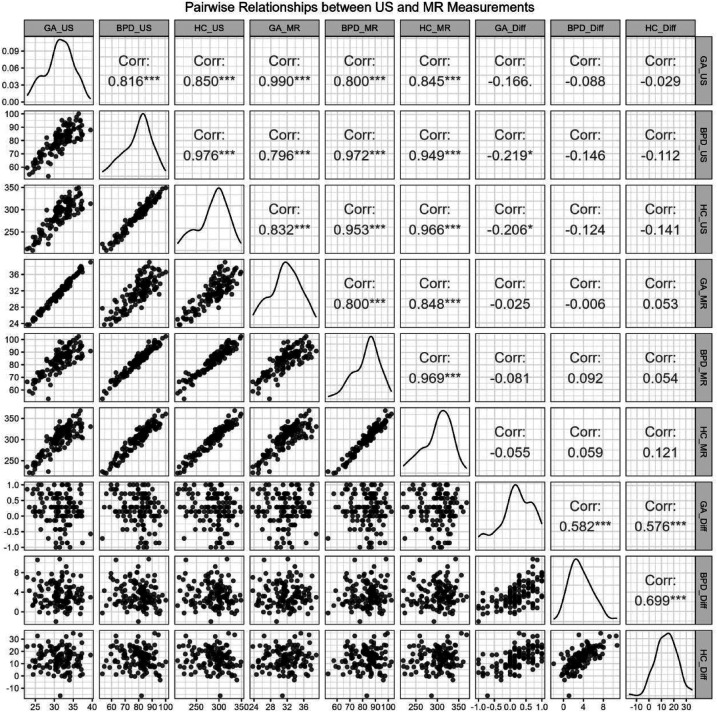
A summary matrix displaying pairwise correlation coefficients and scatterplots for: GA, HC, and BPD from each examination type; and the differences in GA, HC, and BPD between the two examination types.

**Figure 4:**
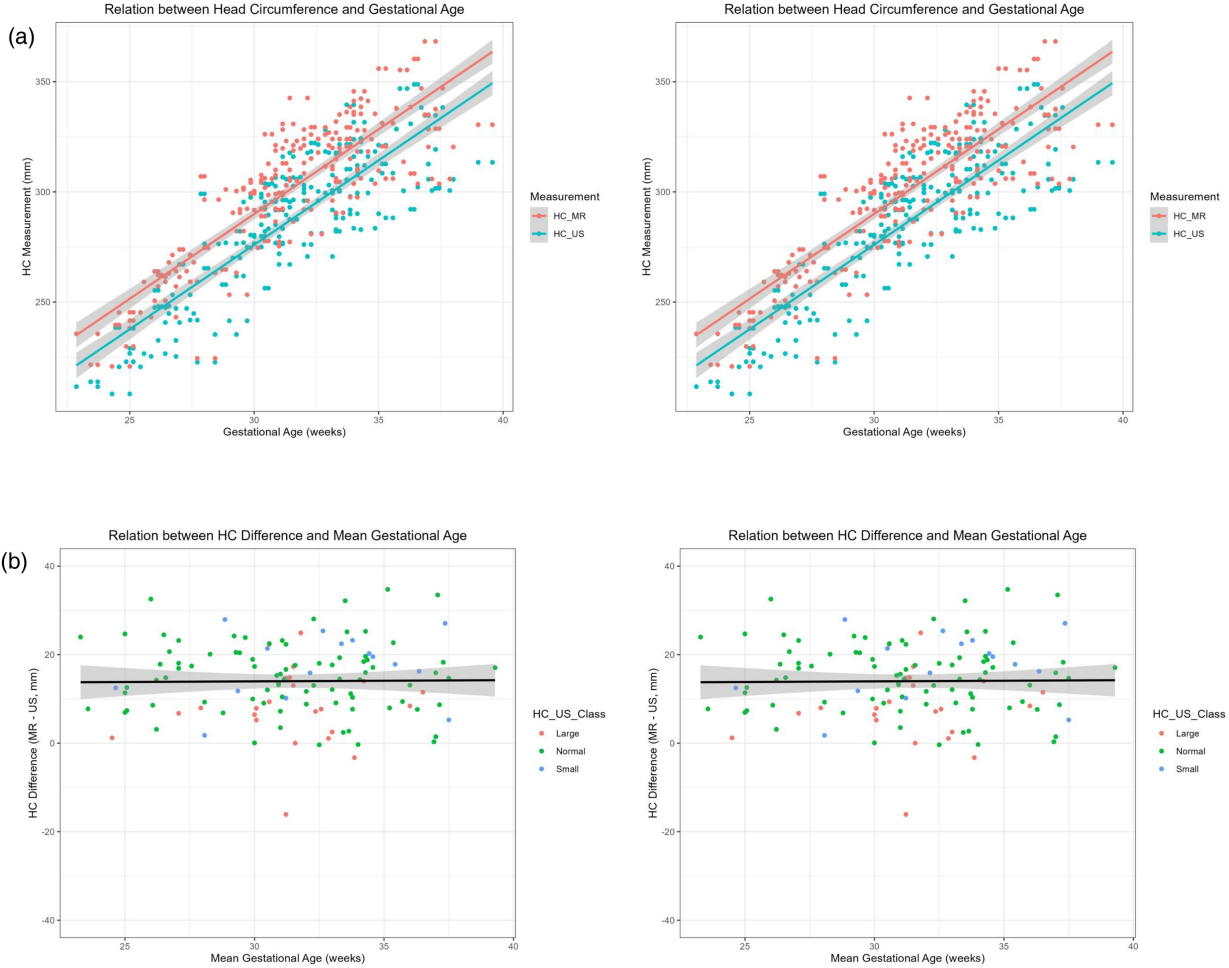

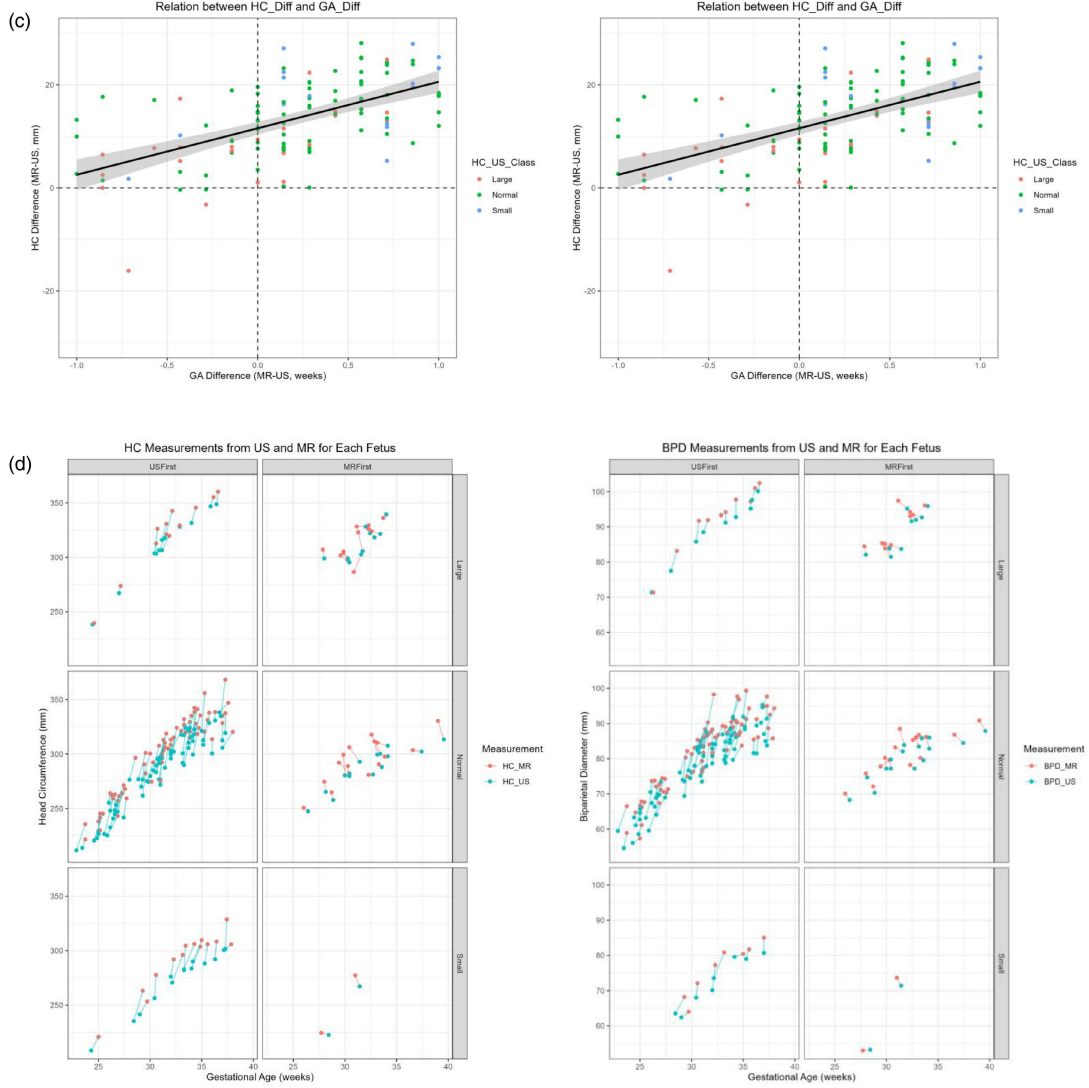
**(a)** Correspondence of HC and BPD with gestational age. Red indicates MR measurement results; blue indicates US-reported results. **(b)** Correspondence of the discrepancy between MR and US results (for HC/BPD) with the average gestational age (taken as the mean of the MR and US examination dates, to account for potential timing differences between the two scans). The data are categorized according to US diagnostic criteria: red for HC/BPD ≥97th percentile (large), green for HC/BPD between the 3rd-97th percentiles (normal), and blue for HC/BPD <3rd percentile (small). **(c)** Correspondence of the discrepancy between MR and US results (for HC/BPD) with the time interval between the MR and US examinations. Color categorization follows the same US diagnostic criteria as in Figure 4b. **(d)** HC and BPD of each fetus plotted against the gestational age at the time of each individual examination (MR or US). The data are compared based on the sequence of MR/US scans and US diagnostic outcomes. Red data points represent MR examinations; blue data points represent US examinations.

Table 3 and Figure 3 summarize the relationship between GA and biometric measurements obtained by US and MRI. Figures 4a,b show strong positive correlations between GA and both HC and BPD for each modality (US: HC *r* = 0.850, BPD *r* = 0.816; MRI: HC *r* = 0.848, BPD *r* = 0.800; all *P* < 0.001). Across 25–40 weeks’ gestation, MRI values were systematically larger than US values, reflected by the MRI regression lines lying consistently above the US lines. Figure 4c plot the measurement differences (MRI−US) against the GA differences (MRI−US). The discrepancies showed mild, significant trends for both HC (*r* = 0.576, *P* < 0.001) and BPD (*r* = 0.582, *P* < 0.001).

**Table 3:** Correlation between gestational age and biometric measurements obtained by US and MRI.

Measurements	GA_US	BPD_US	HC_US	GA_MR	BPD_MR	HC_MR	GA_Diff	BPD_Diff	HC_Diff
GA_US	/	0.816	0.850	0.990	0.800	0.845	−0.166	−0.088	−0.029
BPD_US	0.816	/	0.976	0.796	0.972	0.949	−0.219	−0.146	−0.112
HC_US	0.850	0.976	/	0.832	0.953	0.966	−0.206	−0.124	−0.141
GA_MR	0.990	0.796	0.832	/	0.800	0.848	−0.025	−0.006	0.053
BPD_MR	0.800	0.972	0.953	0.800	/	0.969	−0.081	0.092	0.054
HC_MR	0.845	0.949	0.966	0.848	0.969	/	−0.055	0.059	0.121
GA_Diff	−0.166	−0.219	−0.206	−0.025	−0.081	−0.055	/	0.582	0.576
BPD_Diff	−0.088	−0.146	−0.124	−0.006	0.092	0.059	0.582	/	0.699
HC_Diff	−0.029	−0.112	−0.141	0.053	0.054	0.121	0.576	0.699	/

Figure 4d further stratify paired measurements by examination sequence (US-first vs. MRI-first) and by US-based fetal size classification (large, normal, small). Within each stratum, MRI values were almost invariably higher than US values, with the largest discrepancies observed in the normal and large subgroups. These data confirm a systematic MRI overestimation that is independent of imaging sequence or initial biometric category.

### 3.3 Linear regression models from MR measurements to predict US measurements

Linear regression analyses were performed to predict US-derived head circumference (HC_US) and biparietal diameter (BPD_US) from their corresponding MRI measurements (HC_MR or BPD_MR), MRI gestational age (GA_MR), the GA difference between MRI and US (GA_Diff = GA_MR−GA_US), and the interaction term GA_MR × GA_Diff. Both models explained more than 95% of the variance (HC: adjusted *R*^2^ = 0.957, *F*_4,119_ = 685.9, *P* < 0.001; BPD: adjusted *R*^2^ = 0.966, *F*_4,119_ = 880.3, *P* < 0.001). In each model, the MRI measurement was the dominant predictor (HC_MR: *β* = 0.903 mm mm^−1^, SE = 0.036, *P* < 0.001; BPD_MR: *β* = 0.910 mm mm^−1^, SE = 0.028, *P* < 0.001), while GA_MR alone was not significant (HC: *β* = 0.361 mm week^−1^, *P* = 0.284; BPD: *β* = 0.125 mm week^−1^, *P* = 0.137). GA_Diff exerted a significant negative effect (HC: *β* = –34.98 mm week^−1^, *P* = 0.005; BPD: *β* = –10.86 mm week^−1^, *P* = 0.001), and the interaction term GA_MR × GA_Diff was also significant (HC: *β* = 0.793 mm week^−2^, *P* = 0.040; BPD: *β* = 0.253 mm week^−2^, *P* = 0.017), indicating that the impact of GA_Diff increases with advancing GA. Residual diagnostics revealed no violations of normality or homoscedasticity for either model.

**Figure 5:**
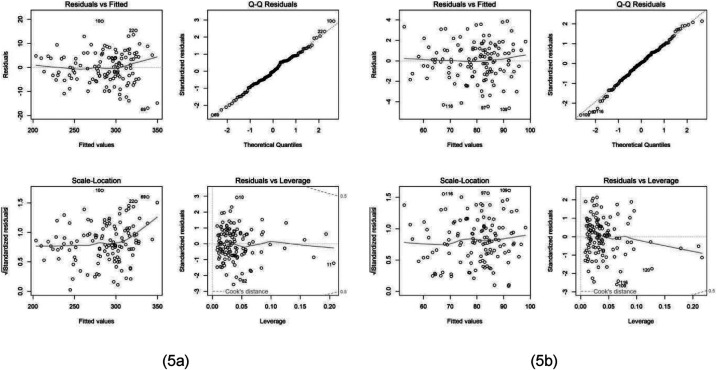
Diagnostic plots for the linear regression model of HC **(a)** and BPD **(b)**. The residuals vs. fitted values plot (top left) assesses linearity and reveals no strong systematic pattern, with a mild curvature at higher fitted values. The Q–Q plot of standardized residuals (top right) shows approximate normality, with slight deviations at the tails. The scale–location plot (bottom left) evaluates homoscedasticity and suggests a modest increase in residual variance with increasing fitted values. The residuals vs. leverage plot (bottom right) identifies observations with higher leverage; none exceed conventional Cook's distance thresholds, indicating no highly influential points.

Model diagnostics confirmed that the linear assumptions underlying both regression equations were satisfied (Figure 5). Shapiro–Wilk tests indicated that the residuals for HC (*W* = 0.994, *P* = 0.879) and BPD (*W* = 0.990, *P* = 0.515) were normally distributed. Breusch–Pagan tests detected no evidence of heteroskedasticity (HC: BP = 7.90, *P* = 0.095; BPD: BP = 7.65, *P* = 0.106), and Durbin–Watson statistics were close to 2 (HC: 2.02, *P* = 0.489; BPD: 1.98, *P* = 0.407), ruling out first-order autocorrelation. RESET tests failed to reject the null hypothesis of correct functional form (HC: *F* = 1.00, *P* = 0.369; BPD: *F* = 0.35, *P* = 0.708). Variance inflation factors for the individual predictors were below 4, whereas the interaction term (GA_MR × GA_Diff) and the raw GA_Diff variable exhibited inflation >100, as expected for a product term; nevertheless, collinearity did not destabilize coefficient estimates. Taken together, the models demonstrated adequate validity and reliability for inferential purposes.

### 3.4 Classification efficacy of the MR measurements and its predicted values (USpred)

**Figure 6:**
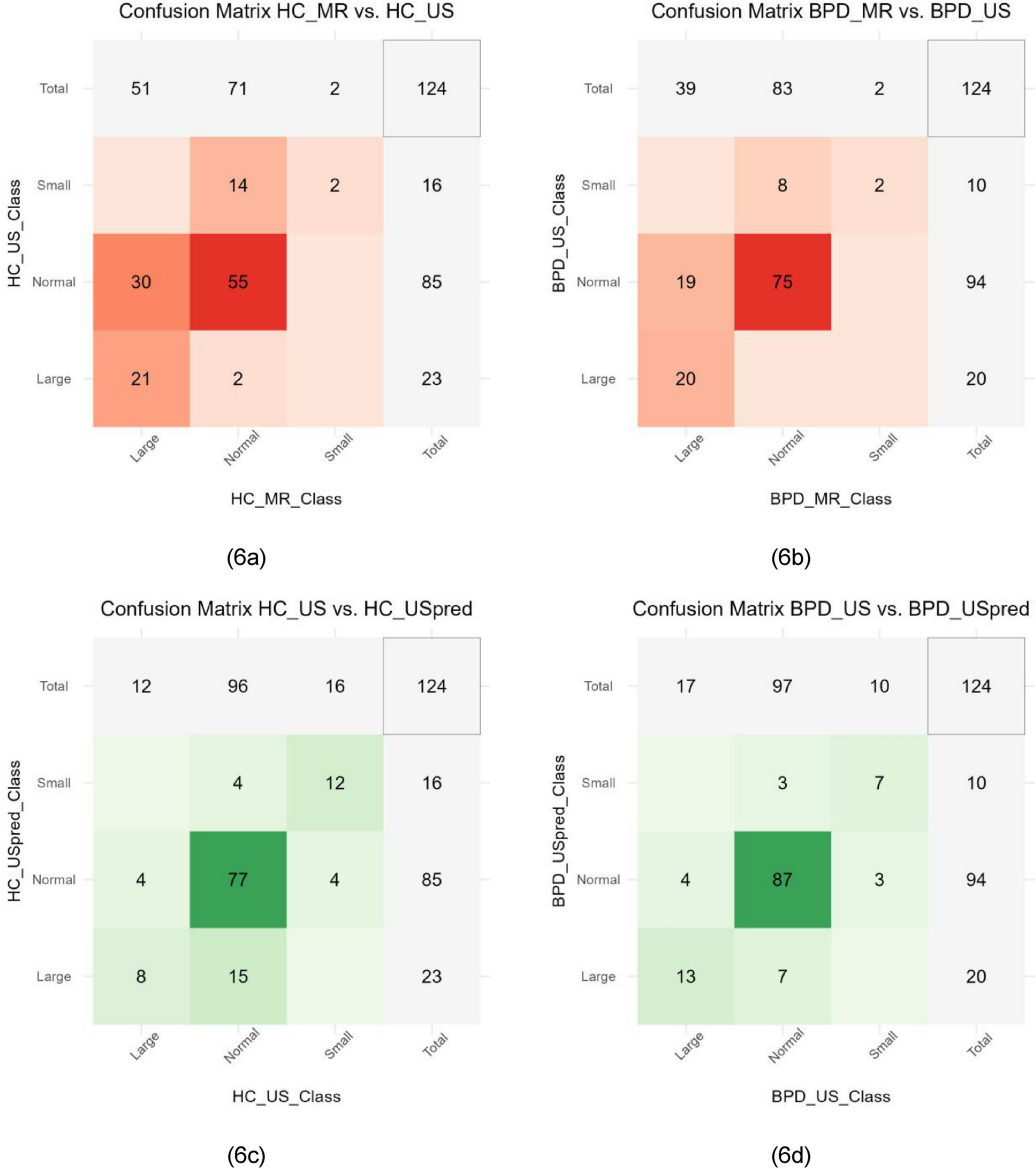
Classification confusion matrices for HC and BPD, comparing: (1) US-reported results vs. MR measurement results **(a,b)**, and (2) US-reported results vs. predictions from the US_pred model **(c,d)**.

Table 4 and Figure 6 present the classification efficacy of MRI measurements and US-predicted classifications (USpred) compared to actual US-based classifications (US_Class) as the reference standard for HC and BPD, categorized into large, normal, and small groups across a cohort of 124 fetuses. Overall, USpred demonstrated superior performance over MR, with higher Matthews Correlation Coefficient (MCC), overall accuracy, sensitivity, specificity, positive predictive value (Pos Pred Value), negative predictive value (Neg Pred Value), F1 score, and Kappa values. For HC, USpred achieved an overall MCC of 0.512 and accuracy of 0.782, compared to MR's 0.322 and 0.629, respectively, with notable improvements in sensitivity (0.668 vs. 0.729) and specificity (0.812 vs. 0.764). Subgroup analysis revealed USpred's enhanced sensitivity for large HC (0.348 vs. 0.412) and normal HC (0.906 vs. 0.775), though MR showed higher specificity for large HC (0.973 vs. 0.960). For small HC, USpred maintained perfect sensitivity (1.000 vs. 0.750) and improved specificity (0.963 vs. 0.885). For BPD, USpred also outperformed MR, with an overall MCC of 0.639 and accuracy of 0.863 compared to 0.528 and 0.782, respectively, and better-balanced sensitivity (0.759 vs. 0.666) and specificity (0.867 vs. 0.850). Subgroup results for BPD showed USpred's improved sensitivity for large (0.65 vs. 1.000) and normal (0.926 vs. 0.798) classifications, while MR excelled in specificity for small BPD (1.000 vs. 0.974). These findings indicate that USpred provides a more reliable classification of fetal HC and BPD sizes compared to direct MR measurements against the US standard.

**Table 4:** Classification efficacy of MRI measurements and USpred compared to actual US-based classifications.

Class	MCC	Overall accuracy	Sensitivity	Specificity	Pos Pred Value	Neg Pred Value	F1	Kappa
HC								
MR vs. US								
Overall	0.322	0.629	0.729	0.764	0.562	0.764	0.498	0.278
Large			0.412	0.973	0.913	0.703	0.568	0.419
Normal			0.775	0.434	0.647	0.59	0.705	0.216
Small			1.000	0.885	0.125	1.000	0.222	0.199
USpred vs. US								
Overall	0.512	0.782	0.668	0.812	0.740	0.848	0.686	0.515
Large			0.348	0.96	0.667	0.866	0.457	0.378
Normal			0.906	0.513	0.802	0.714	0.851	0.453
Small			0.75	0.963	0.75	0.963	0.750	0.713
BPD								
MR vs. US								
Overall	0.528	0.782	0.666	0.85	0.805	0.824	0.62	0.459
Large			1.000	0.817	0.513	1.000	0.678	0.591
Normal			0.798	0.733	0.904	0.537	0.847	0.472
Small			0.200	1.000	1.000	0.934	0.333	0.315
USpred vs. US								
Overall	0.639	0.863	0.759	0.867	0.787	0.883	0.771	0.646
Large			0.65	0.962	0.765	0.935	0.703	0.651
Normal			0.926	0.667	0.897	0.741	0.911	0.613
Small			0.700	0.974	0.700	0.974	0.700	0.674

## 4 Discussion

### 4.1 Main findings

This study demonstrated that, between 25 and 40 weeks of gestation, fetal MRI measurements of HC and BPD systematically overestimate values compared with US measurements. This systematic difference was observed regardless of examination sequence and initial US-based biometric classification. Both modalities showed strong positive correlations between gestational age and HC/BPD, confirming the biological consistency of fetal head growth measurements across imaging methods.

To address the practical issue that US and MRI examinations are often performed on different dates, we developed linear regression models incorporating MRI measurements, MRI gestational age, GA_Diff, and the interaction term GA_MR × GA_Diff. The models showed high goodness of fit and satisfactory diagnostic performance. When evaluated against US-based clinical classification standards, USpred values provided more reliable classification than direct MRI measurements.

### 4.2 Novelty and distinguishing contributions

The present study has several distinguishing contributions compared with previous work.

First, instead of merely reporting correlations between MRI and US measurements, this study performed paired individual-level comparisons between the two modalities. This is important because a high correlation does not necessarily indicate agreement. Our results showed that although MRI and US measurements were strongly correlated, MRI systematically overestimated both HC and BPD.

Second, this study considered the real-world clinical interval between US and MRI examinations. Previous studies often ignored this issue or treated the two modalities as if they were obtained at exactly the same gestational time point. By introducing GA_Diff and its interaction with GA_MR into the regression model, the present study accounted for the potential influence of fetal growth during the examination interval.

Third, rather than proposing an isolated MRI reference range, this study developed an US-equivalent prediction model. This approach is clinically practical because US-based fetal biometric standards remain the most widely used reference system in prenatal care. The proposed model enables MRI-derived values to be translated into values that are more compatible with existing US-based clinical thresholds.

Fourth, this study evaluated not only numerical agreement but also clinical classification performance. The comparison of direct MRI classification and USpred classification demonstrated that the regression-based correction improved accuracy, MCC, Kappa, and F1-score. This suggests that the model may reduce misclassification when MRI measurements are interpreted using US-derived standards.

### 4.3 Comparison with previous studies

Previous studies have reported that fetal MRI can provide accurate anatomical visualization and may be particularly valuable when US is technically limited. Some studies have attempted to establish MRI-based fetal biometric references, but the lack of standardized MRI measurement protocols and the limited clinical adoption of MRI-specific reference ranges restrict their routine application. Other studies have compared fetal biometric parameters between MRI and US, but many have focused mainly on correlation rather than agreement or have not incorporated examination interval into the analysis (25).

The present findings are consistent with the concept that MRI and US are not interchangeable without adjustment. The systematic overestimation observed in MRI may be related to differences in imaging plane selection, tissue contrast, boundary definition, measurement landmarks, and post-processing methods. For example, MRI provides clearer delineation of cranial structures in certain settings, but its measurement plane and contour-based calculation may differ from routine sonographic measurement conventions. These methodological differences may partly explain why MRI-derived HC and BPD values are larger than US-derived values.

### 4.4 Clinical implications

The proposed USpred model has practical implications for prenatal imaging assessment. In clinical practice, fetal MRI is frequently performed as an adjunct to US, especially when fetal central nervous system abnormalities are suspected. However, clinicians often interpret MRI biometric results using US-derived reference standards because MRI-specific standards are not universally available. Direct application of US standards to MRI values may result in overdiagnosis of large HC or BPD.

By converting MRI measurements into US-equivalent values, the proposed model may improve the consistency of MRI-based interpretation with established US standards. This is particularly useful in multidisciplinary prenatal counseling, where US and MRI findings are often jointly reviewed. The model may also be helpful for longitudinal assessment when US and MRI are performed at different time points.

### 4.5 Limitations

This study has several limitations. First, it was a single-center retrospective study with a moderate sample size. Although the cohort included 124 fetuses, external validation using independent multicenter datasets is needed before broader clinical application. Second, MRI examinations were performed using different scanner models and field strengths. While this reflects real-world practice, scanner-related variability may influence measurement consistency. Third, US measurements were extracted from clinical reports rather than remeasured retrospectively from stored images, which may introduce operator-related variability. Fourth, although the MRI measurement process was semi-automated and reviewed by a senior radiologist, manual contour correction may still introduce observer variability. Future studies should assess interobserver and intraobserver reproducibility in more detail. Fifth, the proposed regression model was developed using fetuses between 25 and 40 weeks of gestation and may not be directly applicable to earlier gestational ages. Finally, the study used US-based standards as the reference for classification because of their clinical predominance; however, US itself is not a perfect gold standard.

### 4.6 Future directions

Future studies should validate the proposed US-equivalent prediction model in larger, prospective, multicenter cohorts. Additional work is also needed to evaluate whether model performance differs according to fetal position, maternal body habitus, MRI scanner type, magnetic field strength, and fetal abnormality subtype. Fully automated segmentation and measurement algorithms may further improve reproducibility and reduce processing time. Ultimately, integration of MRI-to-US conversion models into clinical imaging workstations may facilitate standardized fetal MRI biometry assessment.

### 4.7 Conclusion

Fetal MRI measurements of HC and BPD systematically overestimate values compared with US measurements. This discrepancy is present across fetal head-size categories and is influenced by the time interval between examinations. The proposed US-equivalent prediction models effectively correct MRI-related measurement bias and improve agreement with US-based clinical classification standards. These findings support the use of regression-adjusted MRI biometry as a practical tool for more accurate prenatal assessment of fetal head development.

## Data Availability

The original contributions presented in the study are included in the article/Supplementary Material, further inquiries can be directed to the corresponding author/s.
